# Supplementary material for: Patterns of Intron Gain and Loss in Fungi
Source: PLoS Biol. 2004 Nov 30;2(12):e422. doi: 10.1371/journal.pbio.0020422 (PMC532390; doi:10.1371/journal.pbio.0020422)
Supplement: Table S1 — Also available at http://genes.mit.edu/NielsenEtAl/. (4.3 MB ZIP). [file pbio.0020422.st001.zip › NielsenEtAl/html/365.html]

AN3113.1.NCU01826.1.MG06694.1.FG05998.1


```
 CLUSTAL W (1.82) Multiple Sequence Alignments - Introns Inserted


Sequence 1: NCU01826.1	399 aa
Sequence 2: MG06694.1	361 aa
Sequence 3: FG05998.1	409 aa
Sequence 4: AN3113.1	400 aa
Alignment Length: 412 aa
Number Identitical Residues: 176 aa
Alignment Score (without introns) 8807


MG06694.1 	---------------------------MADK~LN------RKALAVD~----SWLWRESS
NCU01826.1	-MGVEEKSRASGDSPRDA--VLPTVNPEAQK~AQPPKSAIHPAFYVS2----IWIAMSSS
FG05998.1 	-MSADEKIRVSGETPRSTSPVLPTVNPGLEN2NFYPRIDFCDATFAN~GARSAWIGFSSS
AN3113.1  	MSSSEEKVRTSGEVSRPE-PTLPTVNPAVDK~PEPPKPTFHPAVYVT2----SWIALSSS
          	  . ...   :.. .    .  .: ..  ::    .      *  .       *:  .**

MG06694.1 	RPGY--------DAN1YPVLLTTYHLTFATVATQLLARYTTLLDGRKTVKMNGQVYMRAI
NCU01826.1	VILFNKWILSAKGFD1FPVVLTTYHLAFSTIMTQILARYTTLLDGRKTVKMTGKVYLRAI
FG05998.1 	VILFNKWLLDTLNFR1YPVILTTYHLTFSTIITQVMARWTPYLDGRKTVKMTARVYIRAV
AN3113.1  	VILFNKHILDYAQFR1FPIILTTWHLAFATFMTQVLARTTTLLDGRKTVKMTGRVYLRAI
          	   :..   .      :*::***:**:*:*. **::** *. *********..:**:**:

MG06694.1 	VPIGFMFSLSLICGNLTYLYLSVAFIQMLK0ATTPVAVLLSSWVLGVAQPNLRIFLNVSV
NCU01826.1	VPIGFFFSLSLICGNLTYLYLSVSFIQMLK0ATTPVAVLLSGWALGVSQINMRVFLNVSV
FG05998.1 	VPIGIFFSLSLICGNLTYLYLSVAFIQMLK~ATTPVAVLISGWILGVSAPNLKQFLNVSA
AN3113.1  	VPIGLFFSLSLICGNVTYLYLSVAFIQMLK0ATTPVAVLLATWGMGMAPVNLKVLTNVSV
          	****::*********:*******:****** ********:: * :*::  *:: : ***.

MG06694.1 	IVVGVVIASFGEIKFVLAGFLFQIGGVIFEALRLTMVQRLLSSAEYKMDPLVSLYYFAPV
NCU01826.1	IVVGVVIASYGELEFVWLGVILQIAGVAFEALRLTMVQRLLSSAEFKMDPLVSLYYFAPI
FG05998.1 	IVVGVIIASMGEIHFVTVGVLFQMGGIIFEALRLTMVQRLLSSADYKMDPLVSLYYFAPI
AN3113.1  	IVFGVIIASFGEIKFVFIGFLFQIAGIIFEATRLVMVQRLLSSAEYKMDPLVSLYYFAPV
          	**.**:*** **:.**  *.::*:.*: *** **.*********::*************:

MG06694.1 	CAAMNATVALFWEMPKVSLAEVYHVGLFNFFLNGLCAFMLNVSVVLL0IGKTSSLVLTIC
NCU01826.1	CALMNGVVALFWEVPRLTMADVDRVGLFYFFLNGLCAFGLNVSVVFL0IGKTSSLVLTLC
FG05998.1 	CAVMNGVVALIWEVPNCTMAEVYHVGLFTFFLNGLCAFMLNVSVVFL0IGKTSAVVLTLC
AN3113.1  	CAVMNGVTALFLEVPTLTMDHIYNVGVWTLLANAMVAFMLNVSVVFL0IGKTSSLVMTLC
          	** **...**: *:*  :: .: .**:: :: *.: ** ******:* *****::*:*:*

MG06694.1 	GVLKDVLLVAASVIIWPDKPVTGLQLFGYSIALAGMVHYKLGYDALKGYAAEGGRQWAEF
NCU01826.1	GVLKDVLLVVASMVIYG-SQVTLTQFFGYSIALGGMVYYKLGAETIKSYAGEAGRQWADF
FG05998.1 	GVLKDILLVGASMMIWG-TQVSPLQFFGYSIALGGMVYYKLGYEQLKGHIADANRQWADF
AN3113.1  	GVLKDILLVVASMVIWN-TPVTALQFFGYSIALIGLVYYKLGGDKIKEYTSQANRAWAEY
          	*****:*** **::*:  . *:  *:******* *:*:**** : :* : .:..* **::

MG06694.1 	GVRRPVLRRV-LILGSVFTLGIFVLSGLAP-YATEYDATKVLSKAANKVGLPQ----
NCU01826.1	GNRRPVLRRISIILLSVFVV-FSLLGGFAPNYAAGIDPTEYLNEAKSKIGMSA----
FG05998.1 	GARKPVLRKVTVLVATAFLI-FTLFGGFAPSYSSEVDPATLANEVSNRFGINDAQHV
AN3113.1  	GATHPAQRRF-VIIGAALLSFFLLVGSMAPSYAPDSVANVKGMLGGASTGNA-----
          	*  :*. *:. ::: :.:   : :...:**.*:.   .           *
```
